# The anti‐inflammatory effects of equine bone marrow stem cell‐derived extracellular vesicles on autologous chondrocytes

**DOI:** 10.1002/vro2.22

**Published:** 2021-11-10

**Authors:** William Edward Hotham, Charlotte Thompson, Lin Szu‐Ting, Frances Margaret Daphne Henson

**Affiliations:** ^1^ Division of Trauma and Orthopaedic Surgery University of Cambridge Cambridge Cambridgeshire UK; ^2^ Cambridge Veterinary School Cambridge Cambridgeshire UK

**Keywords:** chondrocytes, extracellular vesicles, inflammation, lameness, osteoarthritis, stem cell therapy

## Abstract

**Background:**

Osteoarthritis (OA) in the horse is an economic and welfare issue and there are no current disease modifying drugs available. Stem cells have been suggested as a therapeutic intervention for OA, originally on the basis of their regenerative capacity. However, it is hypothesised that mesenchymal stem cells (MSC) exert their effects via paracrine factors including the production of extracellular vesicles that can themselves recapitulate the MSC effects in the joint.

**Objectives:**

To isolate extracellular vesicles from bone marrow MSC and investigate their anti‐inflammatory effects on chondrocytes.

**Study design:**

An in vitro assessment of the effect of direct culturing extracellular vesicles on artificially inflamed chondrocytes.

**Methods:**

Extracellular vesicles were isolated from bone marrow MSC using differential sequential ultracentrifugation. Vesicles were characterised using electron microscopy, nanoparticle tracing analysis and protein analysis. Vesicle internalisation was carried out via vesicles being pre‐stained and co‐cultured with equine chondrocytes before analysis using confocal microscopy. The effects of vesicles on artificially inflamed chondrocytes was examined using quantitative PCR.

**Results:**

To the best of the authors' knowledge, this is the first study to isolate and characterise extracellular vesicles from equine bone MSC. Vesicles were taken up by autologous chondrocytes and had anti‐inflammatory effects on gene expression following chondrocyte exposure to tumour necrosis factor α and Interleukin 1β.

**Main limitations:**

Only three independent biological repeats were performed and the work was done in vitro.

**Conclusion:**

Extracellular vesicles can be isolated from equine bone marrow MSC; they may be taken up by chondrocytes and have an anti‐inflammatory action.

## INTRODUCTION

The use of mesenchymal stem/stromal/signalling cells (MSC)^1^ to treat orthopaedic diseases in horses has become widespread in the last decade^2^, ^3^ with tendon/and ligament injuries, synovitis, osteoarthritis (OA) and cartilage lesions all being considered as candidates for MSC therapy. Whilst the majority of MSC therapies have been performed using autologous cells, the increasing commercialisation of stem cell therapies has led to the launch of allogeneic MSC products.^4^

In the horse OA is a major cause of lameness and poor performance. Characterised by changes in the joint including synovitis and subchondral bone abnormalities, it leads to degeneration of articular cartilage.^5^ Treatment of OA is challenging, with no disease modifying therapies available. Non‐steroidal anti‐inflammatory drugs are commonly given to alleviate signs such as pain and inflammation; however, they do not restore the cartilage to its previous condition. The use of MSC to treat equine OA has increased in recent years and whilst intra‐articular MSC are considered to be safe to administer, it is well recognised that, in the horse, these injections can cause transient inflammation and synovitis.^6^ There are several different sources of bone marrow MSC in the body including from various limbs; however, the anatomical location of these tissues does not affect the function of the cells.^7^, ^8^ Additionally, there are other complications and disadvantages of using MSC including limited cell survival, immune‐rejection, senescence‐induced genetic instability or loss of function and the theoretical risk of malignant transformation.^9^, ^10^

The original theories to explain the beneficial effects of MSC proposed that their mechanism of action was primarily to engraft and regenerate damaged tissue. However, it has become clear that this ‘engraftment and build’ concept is not supported by evidence. It has now been demonstrated that MSC exert their effect via paracrine actions through the secretion of anti‐inflammatory and regenerative factors;^11^, ^12^ that many of these paracrine actions have been demonstrated to reside in small, membrane bound particles, extracellular vesicles (EV), secreted by the MSC.^13^

Extracellular vesicles are secreted by all cells and are involved in cell–cell communication;^14^ they are sub‐classified depending on their size into exosomes (40–100 nm), microvesicles (100–1,000 nm) and apoptotic bodies (100–5,000 nm).^15^ They act as a paracrine signalling system, stably transporting proteins, RNAs [rRNA, tRNA, miRNA, lnc(RNA)] and mitochondrial DNA through the extracellular environment.^16^, ^17^ Extracellular vesicles attach to the recipient cell membrane and are internalised, exerting their effects by direct ligand–receptor interactions on the cell surface or by intracellular effects of their cargo including affecting gene expression through de novo translation and post‐translation regulation of target mRNAs.^18^ Thus, EV can theoretically recapitulate the therapeutic effects of their parent cell.^16^, ^19^ The characterisation of human EV has been extensively reported.^11^ The identification of human cells based on their protein characterisation is considered good practice. In commonly used non‐human MSC sources, such as mouse and rat, such protein characterisation has not routinely been performed.^20^

In vivo, bone marrow derived mouse MSC EV have been reported to prevent cartilage breakdown^21^ in the joint and, as cell‐free, minimally immunogenic particles, EV may represent an opportunity for equine joint disease treatment and regeneration. The aims of this study were i) to isolate and characterise EV from horse bone marrow derived MSC (BM‐MSC) and ii) to investigate the effect of these EV on chondrocytes cultured in a pro‐inflammatory environment in vitro.

## METHODS

### Animals

Bone marrow and cartilage was collected postmortem from three adult horses (aged 18‐24 years) (one male, two female) that were euthanased for reasons other than orthopaedic disease with no inflammatory related diseases at the Department of Veterinary Medicine, University of Cambridge, UK with full ethical consent from the owners. The femoro‐patellar joints were dissected and a visual inspection of the cartilage surface carried out to ensure no cartilage abnormalities were detected.

### Cell harvest and culture

Bone marrow was aspirated from the medullary cavity of the distal femur and collected into 2,000 U/ml sterile Heparin (Sigma, UK) diluted in phosphate buffered saline (PBS) (Sigma, UK) at 4°C. Between 5 and 10 ml of bone marrow was collected from each donor. The marrow was passed through a 70 μm cell strainer (Miltenyi, UK), before undergoing two rounds of centrifugation (300 × *g*, 5 min at room temperature). The supernatant was discarded and the pellet re‐suspended in standard culture media. Standard culture media consisted of α‐MEM (Invitrogen, UK), supplemented with 10% fetal calf serum (FCS) (First Link, UK), 1% penicillin/streptomycin (Sigma, UK), 1% Glutamax (Thermofisher, USA), 5 μg/ml ascorbic acid (Sigma, UK) and 50 ng/ml Human Fibroblast Growth Factor Beta (Peprotech, USA). Cells were initially plated in T75 flasks and after a first passage they were then cultured in T175 flasks (Fisher, UK).

Cartilage was removed from the articular surface of the distal femur using sharp dissection and collected into α‐MEM at 4°C. Cartilage fragments were washed in PBS, before being dissected into <1 mm^3^ pieces. The cartilage was then incubated in 0.2% collagenase II (Roche, Germany) dissolved in standard culture media overnight under standard tissue culture conditions (humidified incubator (Sanyo, UK) at 37˚C and 5% CO_2_). The contents of the flask were then filtered (70 μm nylon cell strainer). Of the filtered substrate, any cells were pelleted from solution via centrifugation (300 × *g*, 5 min). The pellet was re‐suspended and plated for cell culture in standard tissue culture medium and under standard tissue culture conditions. Cells were cultured in T175 flasks.

All cells were cultured in a humidified incubator (Sanyo, UK) at 37˚C and 5% CO_2_. Upon reaching 80% confluence, cells were passaged using trypLE (Life Technologies, USA). In brief, culture media was removed and 100 μl/cm^2^ trypLE was added to the culture flask. The cells were incubated with trypLE for 10 min before mild agitation to detach the cells. The cell containing trypLE was then collected and centrifuged at 300 × *g* for 5 min. The supernatant was discarded, the cells re‐suspended in PBS; an aliquot was counted with a haemocytometer before the cells were re‐centrifuged and re‐suspended in an appropriate volume of culture media and the cells plated.

### Tri‐lineage differentiation of MSCs

Upon reaching approximately 80% confluence at passage 2, the cells were passaged as above before being cultured in the appropriate differentiation medium.

### Chondrogenic differentiation

A total of 2 × 10^5^ MSC were centrifuged at 300 × *g* for 5 min to form a pellet and were then cultured in Stempro Chondrogenic differentiation media (Life technologies, UK) for 21 days under standard cell culture conditions with media changes every 3–4 days in a 15 ml polypropylene falcon tube (Greiner Bio‐one, Australia). The pellet was then fixed in 4% paraformaldehyde (Sigma, UK) for 1 h before paraffin embedding, sectioning at 5 μm, staining with Alcian Blue and imaged using light microscopy.

### Osteogenic differentiation

A total of 2 × 10^5^ MSC were plated into a 6‐well plate with standard culture media for 24 h. The media was then removed, and the cells washed once with PBS before being cultured in osteogenic differentiation media [culture media, 3.5 mM β glycerophosphate (Sigma, UK), 100 nm dexamethasone (Sigma, UK)]^22^, ^23^ with media changes every 3–4 days. Cells were cultured in differentiation media for 21 days before the media was removed, and the cells washed once with PBS and fixed with formalin for 1 h. After fixing, the cells were covered with 0.5% (W/V) alizarin red (pH 4.1). The cells were stained for 15 min before the alizarin red was removed and the cells washed in PBS five times before being left to air dry before analysis using light microscopy.

### Adipogenic differentiation

A total of 1 × 10^5^ MSC were cultured in 6‐well plates for 24 h in standard culture conditions before the media was removed, and the cells washed and StemPro Adipogenesis media (Life Technologies, UK) being added to the cells. The media was changed every 3–4 days, and the cells were cultured for 14 days before being fixed with formalin and stained with oil‐red‐o solution (0.1% V/V) for 1 h. The stain was removed and the cells washed with PBS before imaging and analysis under light microscopy.

### Differential sequential ultracentrifugation for extracellular vesicle isolation

**FIGURE 1:**
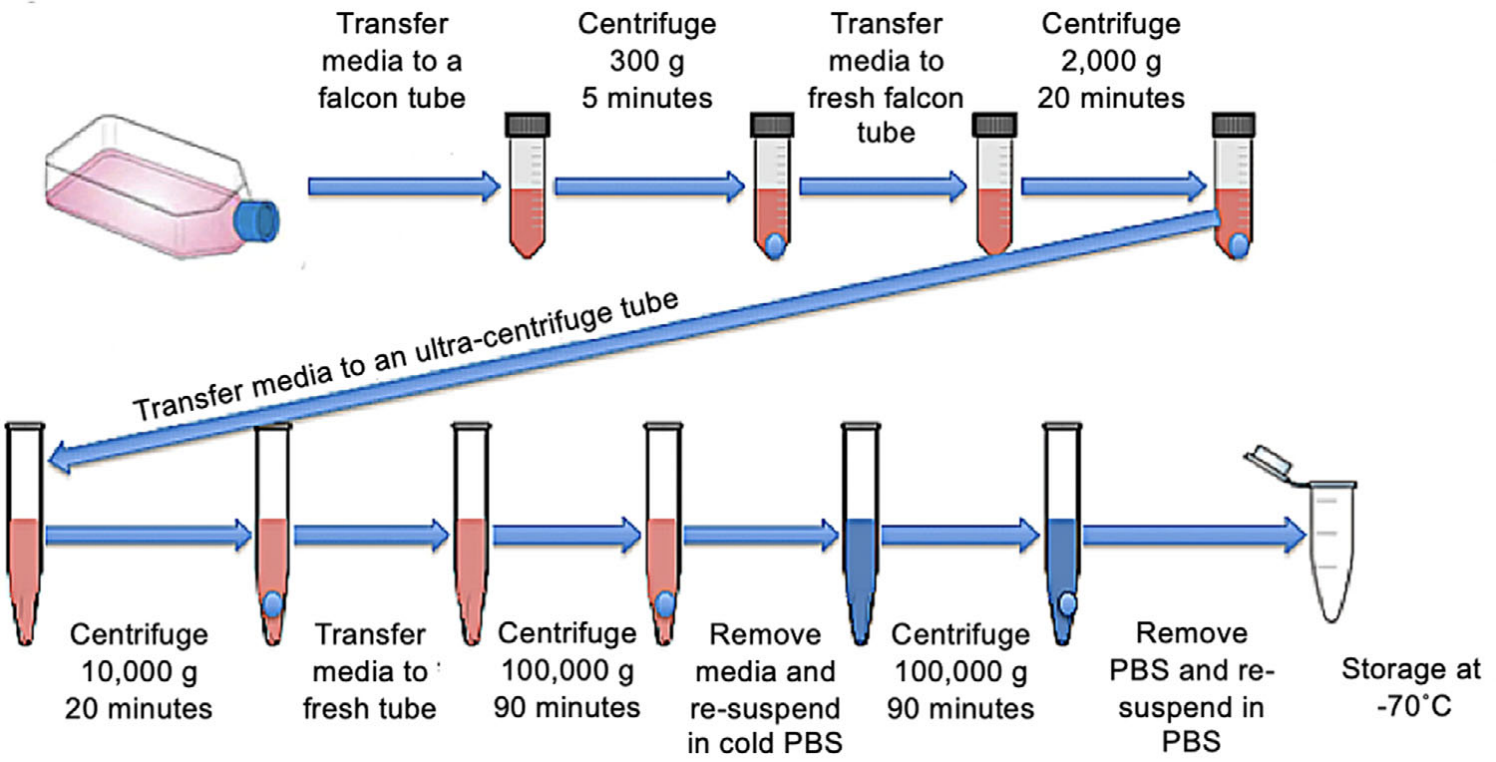
A schematic to demonstrate differential sequential ultracentrifugation for EV isolation from equine bone marrow‐derived MSC. Conditioned media was centrifuged at 300 × *g* for 5 min, 2,000 × *g* for 20 min at 4°C, 10,000 × *g* for 45 min at 4°C, 100,000 × *g* for 90 min at 4°C under vacuum

Extracellular vesicles were isolated from passage 3 BM‐MSC. At passage 3, standard culture media was removed from the cells, which were then washed with PBS and then cultured in FCS free media for 48 h under standard culture conditions. This media was then collected and centrifuged at 300 × *g* for 5 min, before the supernatant was collected and re‐centrifuged at 2,000 × *g* for 20 min. The supernatant was then ultra‐centrifuged using an Optima XPN 100‐K Floor Standing Ultracentrifuge (Beckman, UK) using a SW32 swing bucket rotor (Beckman,UK) at 10,000 × *g* for 45 min. The supernatant was again collected and re‐centrifuged at 100,000 × *g* for 90 min. The supernatant was then discarded and the pellet re‐suspended in PBS and ultra‐centrifuged again at 100,000 × *g* for 90 min at 4°C. The pellet was re‐suspended in PBS and frozen at −70˚C. Figure 1 illustrates the process of EV isolation via differential sequential ultracentrifugation.

### Nanoparticle tracking analysis (NTA)

NTA analysis was performed and analysed using a NanoSight LM10 Nanoparticle Analysis system (Malvern, UK) and NTA 3.2 analytical software as per the manufacturer's instructions. The EV suspension was diluted to give an average particles/frame count of 50 before analysis.

### Transmission electron microscopy

An EV suspension was placed on a ‘glow discharge disk’ prepared by the Cambridge Advanced Imaging Centre (CAIC). The EV loaded disk was then negatively stained with 2% uranyl acetate (Sigma, USA) for 2 min at room temperature, before washing with PBS twice, ready for viewing using TEM. In brief, TEM was performed with a Tecnai G2 TEM (FEI/ThermoFisher) using an accelerating voltage of 200 keV and a 20 μm objective aperture to improve contrast. Images were acquired with an ORCA HR high‐resolution CCD camera with a Hamamatsu DCAM board running Image Capture Engine software, version 600.323 (Advanced Microscopy Technology Corp., Danvers, MA, USA).

### Bicinchoninic acid (BCA) protein assay

The protein content of the EV suspension was measured using the Pierce BCA Protein Assay Kit as per the manufacturer's instructions (Thermoscientific, UK). An aliquot of EV suspension was used to quantify surface protein or added to 20% sodium dodecyl sulphate for 20 min (Sigma, UK) to lyse the EV and quantify total protein.^24^ The percentage surface and internal protein was then calculated.

### Internalisation

Extracellular vesicles from 4 × 10^4^ MSCs were labelled with the fluorescent dye PKH67 (Sigma, UK) via incubation at room temperature for 30 min as per manufacturer's instructions. In brief, the EV were suspended in 994 μl of Diluent C (Sigma, UK) and 6 μl of PKH67. The reaction was then quenched via the addition of 10% Bovine Serum Albumin (BSA) (Sigma, UK). The EV were pelleted via centrifugation at 100,000 × *g* for 90 min and the supernatant containing unbound dye was removed. The pellet was then washed via the addition of 1 ml of 10% BSA and re‐centrifuged to ensure complete quenching and removal of any unbound dye. The supernatant was removed and the pellet was then re‐suspended in PBS and washed once more via this centrifugation process before culture with equine chondrocytes for 1 h under standard culture conditions. After 1 h, cells were fixed with formalin for 1 h before the cell membranes were stained with WGA‐55 (Thermofisher, UK) as per manufacturer's instructions. Cells were permeabilised by the addition of 10% Triton X and 10% BSA in PBS for 20 min before nuclear staining with DAPI. Cells were imaged via confocal microscopy (Leica SP5).

### Effect of extracellular vesicles on chondrocytes grown under inflammatory conditions

A total of 2.5 × 10^5^ equine chondrocytes were cultured in the presence of either Interleukin‐1β (IL‐1β) or tumour necrosis factor‐α (TNF‐α) at concentrations of 1 and 2 ng/ml. After 12 h of culture, EV that had been isolated from 2.5 × 10^5^ MSC by differential sequential ultracentrifugation and re‐suspended in 5–10 μl of room temperature PBS were added to the standard culture medium, equivalent to a 1:1 ratio of chondrocytes:MSC.

After 72 h of culture in the presence of EV, culture media was removed from the cells and 300 μl of RNA lysis buffer (QUIazol, Qiagen, Germany) was added. mRNA extraction was performed using the Direct‐zol mRNA microprep kit as per manufacturer's instructions (Zymo, USA). cDNA synthesis was then performed using a QuantiTect Reverse Transcription Kit (Qiagen, Germany). MMP‐13, ADAMTS4, ACAN and HPRT primers were obtained. Primers sequences (5′ to 3′) are shown in Table 1.

**TABLE 1:** The primer sequences 5′−3′ of the genes analysed using qPCR

Gene	Forward Primer 5′−3′	Reverse Primer 5′−3′
MMP13	GGGAGCACTCATGTTTCCGA	GGGTTCGGGTCTTCATCTCC
ADAMTS4	TGTGGTCACTATTCCTGCGG	TGAGGGCATAGGAGCCATCT
ACAN	GACCACTTTACTCTTGGCGTTTG	GTCAGGGTCTGAAACGTCTACTGA
HPRT‐1	GGCAAAACAATGCAAACCTT	CAGGGCATATCCTACGACAA

The qPCR analysis was performed using a QuantiFast SYBR Green PCR Kit (Qiagen, Germany) in a StepOnePlus Machine (ThermoFisher, UK). Target genes were normalised using the housekeeping gene hypoxanthine‐guanine phosphoribosyltransferase (HPRT‐1) and the relative gene expression was calculated by comparing 2^−ΔCt^ values.

### Statistics

All statistical work was performed using a one‐way ANOVA with a Tukey's multiple comparisons test using GraphPad Prism 9, version 9.0.0 (121). If *p < 0.05, **p < 0.01,***p < 0.001.

## RESULTS

### Bone‐marrow‐derived mesenchymal stem cell differentiation

**FIGURE 2:**
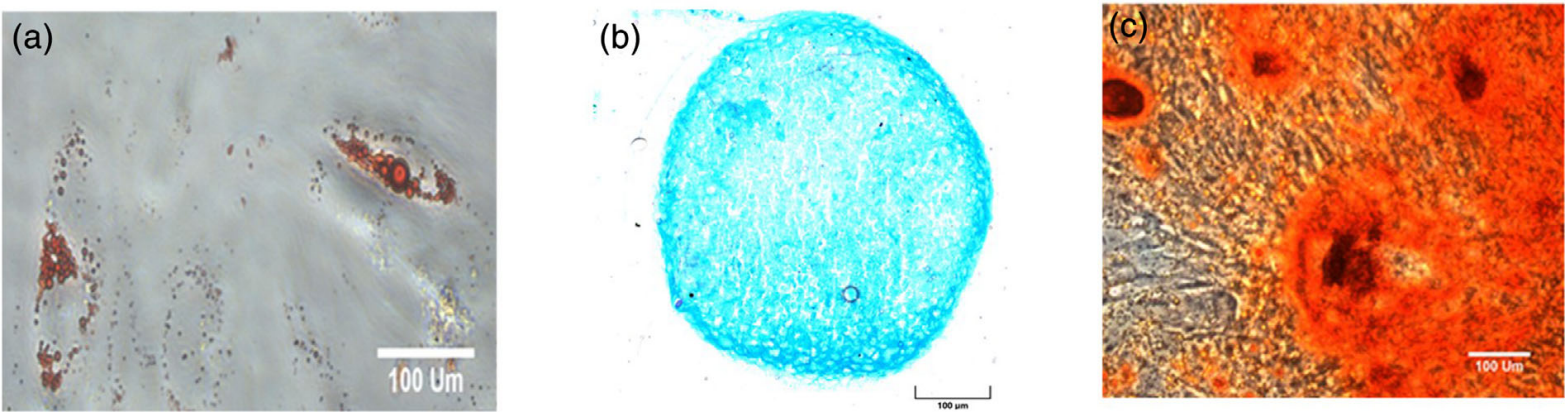
Tri‐lineage differentiation of bone marrow‐derived MSC (BM‐MSC). The BM‐MSC were cultured in adipogenic, chondrogenic and osteogenic media for up to 21 days. (a) Staining with Oil‐Red‐O showing the presence of red fat vacuoles stained red confirming adipogenesis (phase contrast imaging). (b) Staining with Alcian blue showing the presence of blue chondrocytes confirming chondrogenesis (bright field microscopy). (c) Staining with alizarin red showing the presence of red mineral staining confirming osteogenesis (phase contrast microscopy) (*n* = 3) (scale bar = 100 μm)

Cells obtained from bone marrow were demonstrated to be adherent to tissue culture plastic and capable of tri‐lineage differentiation into adipocytes (Figure 2a), chondrocytes (Figure 2b) and osteoblasts (Figure 2c) in the appropriate culture media.

### Extracellular vesicles production, isolation and characterisation

**FIGURE 3:**
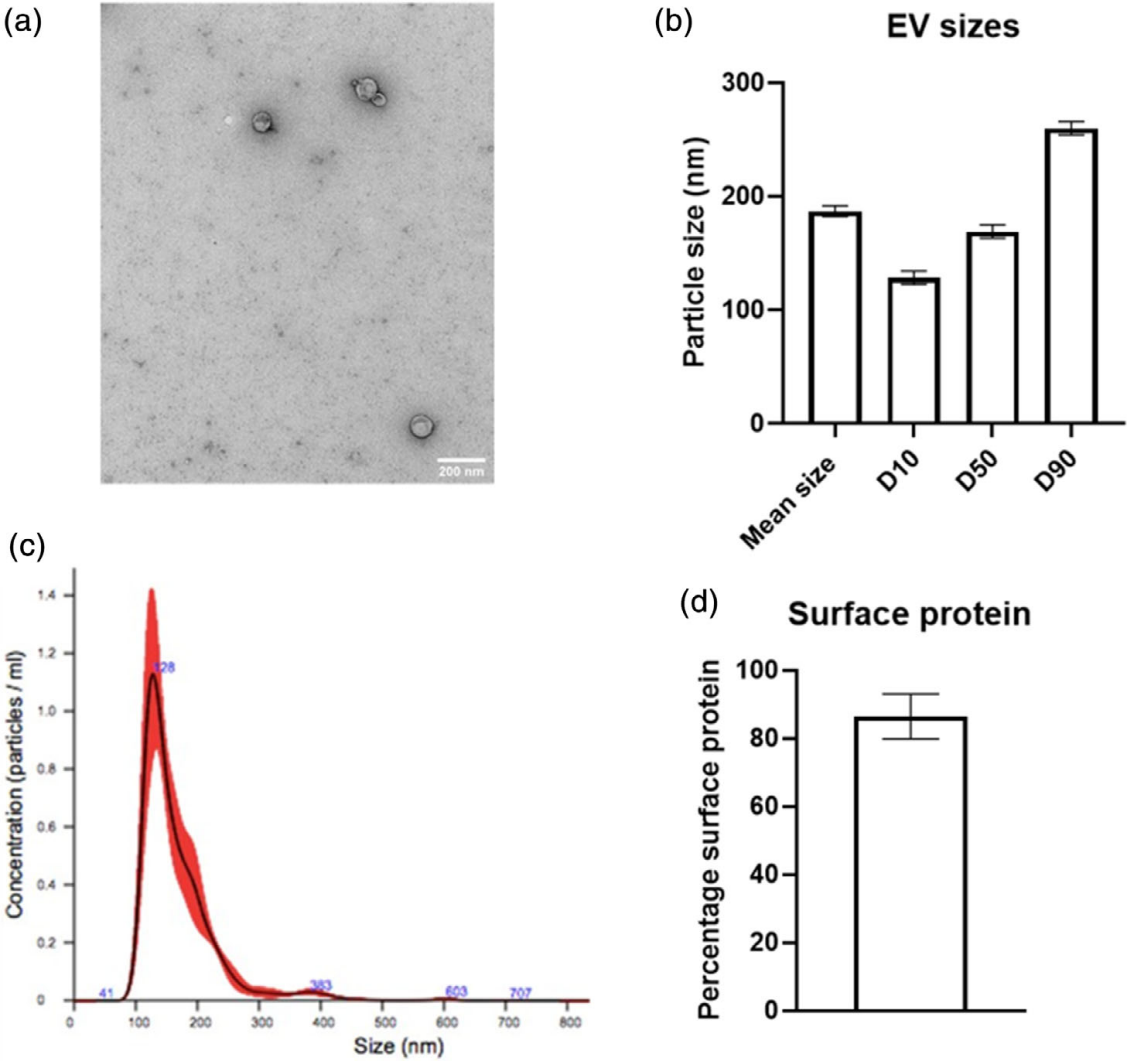
**Extracellular vesicles (EV) characterisation** (a) Transmission electron microscopy showing isolated EVS have a circular morphology and that different EVs have different sizes. Scale bar = 100 nm. (b) Nanoparticle tracking analysis showing the size distribution of the EV population was 45–450 nm characteristic of EV. The size of the EV at the 10th, 50th and 90th percentile is also shows the variation in sizes across the sample population. (c) The mean particle size was 182.48 nm with a heterogeneity of sizes across the sample. (d) Analysis of protein content of the EVs showed that the mean percentage of EV surface protein in comparison to the internal protein is 86% (±7% 1 × SD). Error bars = 1 × SD (*n* = 3)

Extracellular vesicles were obtained from BM‐MSC from three horses via differential sequential ultracentrifugation, with the mean yield of EV 4.61 × 10^10^/million MSCs (±0.89 × 10^10^ = 2 × SD). The EV obtained were characterised in several ways. First, transmission electron microscopy (TEM) was used to directly visualise the EV. TEM confirmed that the particles isolated by differential sequential ultracentrifugation were circular structures with a heterogeneous size, characteristic of EV (Figure 3a). The size of the EV produced by differential ultracentrifugation was measured with NTA. This showed that the mean size of the EV isolated was 186 nm [±3.9 nm (SD)] (Figure 3b), with the size of the EV at the smallest 10th percentile to be 128.3 nm (±4.7 nm) at the 50th percentile 168.9 nm (±4.8 nm) and at the 90th percentile to be 259 nm (±4.8 nm) (Figure 3c). Total protein analysis using a BCA revealed that 86.5% (±6.6%) of the total EV protein content was on the surface of the EV (Figure 3d).

### Extracellular vesicle internalisation

**FIGURE 4:**
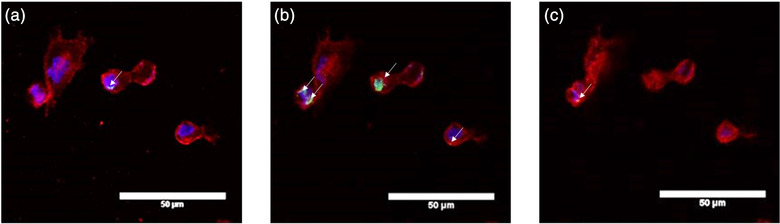
**Confocal imaging of equine chondrocytes showing internalisation of bone marrow derived mesenchymal stem cell extracellular vesicles (MSC‐EV)**. Three images from the confocal z‐stack (top, middle and bottom, respectively) showing EVs pre‐stained with PKH67 (green) and internalised by an equine chondrocyte stained with the membrane stain WGA‐555 (red) and nuclei stain (blue). This figure shows the EV primarily located in the middle of the cell. Scale bar = 50 μm (*n* = 3)

In order to demonstrate that EV were taken up by chondrocytes, confocal microscopy was performed. Autologous chondrocytes were cultured in monolayer and then co‐cultured with or without pre‐labelled EV (Figure 4). Extracellular vesicles were detected within the chondrocytes (Figure 4), confirming that the chondrocytes were able to internalise the EV. The EV were located in the cytoplasm of the chondrocytes.

**FIGURE 5:**
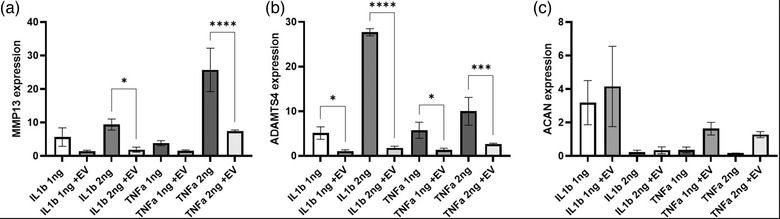
**The effect of extracellular vesicles on chondrocyte MMP‐13, ADAMTS4 and ACAN gene expression**. Gene expression in chondrocytes cultured in the presence of IL‐1β and TNF‐α at concentrations of 1 or 2 ng/ml in the presence and absence of autologous bone marrow MSC‐derived EV. MMP‐13 gene expression is increased in the presence of IL‐1β and TNF‐α in a dose dependant manner. This increase in MMP13 expression is abrogated when EV are applied to the chondrocytes but is only significant at concentrations of 2 ng/ml (*p* < 0.05) (a). Similarly, ADAMTS4 gene expression is increased in the presence of IL‐1β and TNF‐α and as the concentration of both cytokines increases, so does the ADAMTS4 expression. However, upon exposure to autologous BM‐MSC EV, the increase in ADAMTS4 gene expression was lost (b) (*p* < 0.05). Upon exposure to 1 ng/ml of IL‐1β, chondrocyte ACAN gene expression remained higher than any of the other cytokine treatment variables (2 ng/ml IL‐1β, 1/2 ng/ml TNF‐α). Upon exposure to EV and IL‐1β at either concentration, there was no significant difference in ACAN gene expression (c). Upon exposure to TNFα, there was no significant difference in ACAN exposure and a non‐significant increase was observed when EV were applied. Error bars = 1 × SD, ***p < 0.005, **p < 0.01, *p < 0.05. NS = not significant (*n* = 3)

### The effect of bone marrow‐derived MSC extracellular vesicles on chondrocytes cultured under inflammatory conditions

Chondrocytes were cultured under inflammatory conditions and RT‐qPCR performed to assess the effects of culture on gene expression of MMP13 (Figure 5a), ADAMTS4 (Figure 5b) and ACAN (Figure 5c) in the presence of autologous EV‐derived from BM‐MSC. Gene expression was calculated relative to the housekeeping gene HRPT.

In chondrocytes treated with both 1 and 2 ng/ml IL‐1β a dose dependant increase in MMP13 was observed, when cultured in the presence of EV MMP13 levels decreased. This was significant at a IL‐1β concentration of 2 ng/ml. A similar effect was detected when chondrocytes were treated with TNFα, that is, MMP13 levels increased with increasing cytokine concentrations and the presence of EV decreased the expression of MMP13, although a significant decrease in MMP13 expression in the presence of EV was only observed at a concentration of 2 ng/ml of TNFα.

Similar effects were seen with regards to ADAMTS4 gene expression. Increasing cytokine levels caused an increase in ADAMTS4 expression however culture in the presence of EV significantly decreased the expression of ADAMTS4 in all conditions (Figure 5b). The effects of culturing chondrocytes with 2 ng/ml of IL‐1β in comparison to 1 ng/ml caused a large drop in ACAN gene expression similar to levels seen at either concentration of TNFα. The application of EV did not cause any significant change to the chondrocyte ACAN gene expression.

## DISCUSSION

To the best of the authors' knowledge, this study describes for the first time, the isolation and characterisation of EV derived from equine BM‐MSC and demonstrates their ability to alter gene expression of chondrocytes in the presence of IL‐1β and TNF‐α.

The isolation of EV from equine tissue has been previously described, with several studies detailing their isolation from various equine tissues.^25^, ^26^, ^27^, ^28^, ^29^ Of these, four reports obtained the EV from adipose‐derived stem cells and the others were isolated from amniotic stem cells.^25^, ^26^, ^27^, ^29^, ^30^ In these reports a number of techniques were used to isolate EV including ultracentrifugation, size exclusion chromatography and precipitation, all of which led to a yield of EV.^25^, ^26^, ^27^, ^28^, ^29^, ^30^

In this study, we used a differential ultracentrifugation technique to isolate a population of EV from BM‐MSC. This technique allowed the isolation of a population of EV with a mean size of 189 nm. In one study ultracentrifugation, ultrafiltration and charge‐based precipitation techniques were used to isolate an EV population from horse adipose‐derived MSC,^28^ with ultrafiltration giving the highest and ultracentrifugation the lowest yield. This demonstrates that, as in other species, multiple methods can be used to obtain EV. In the study described here, we were able to isolate EV in a reproducible fashion with the EV produced falling within a narrow size range comparable to EV of other species.

The nature of the EV isolated from BM‐MSC was demonstrated using TEM and NTA analysis. The TEM is vital for showing the structure of EV^15^ and, in this study, demonstrated the presence of rounded vesicles of heterogeneous size as previously reported.^5^, ^28^, ^31^, ^32^ The NTA analysis was used to measure the size distribution and the concentration of the isolated EV in the samples studied. Whilst not without some technical limitations, NTA allows for the rapid measurement of these parameters and is widely used in EV studies.^15^ The mean size of the bone marrow‐derived MSC reported in this study (186 nm) is similar to that reported for EV isolated from adipose‐derived MSC in the horse which was variously reported as between 91 and 178 nm depending on the method of isolation^28^ and in other species [mean size 112 nm (murine)^33^ and 231 nm (human)].^34^

No protein characterisation was performed on the EV due to a lack of cross‐reactive antibodies available for this study. This form of characterisation is recommended in the MISEV 2018 criteria for characterising EV; although the criteria are mainly tailored towards EV of human origin.^11^ Future work should investigate equine BM‐MSC EV protein characterisation.

Having isolated EV from equine BM‐MSC, we investigated the interactions between the EV and a target cell in the joint, the chondrocyte. Autologous chondrocytes from normal joints were grown in monolayer culture and incubated with fluorescent labelled EV for one hour. Z‐stack images, acquired with confocal microscopy were used to identify EV in the cellular environment, revealing that the EV were internalised into the cell. In several cells, multiple EV were present and this led to a green cluster being observed. This uptake of BM‐MSC EV has previously been shown in osteoarthritic human chondrocytes.^35^ To the best of the authors' knowledge, it has not been reported before in equine chondrocytes, normal or osteoarthritic. These results confirmed that the isolated EV can be internalised by the potential target cell.

One of the key aims in the treatment of equine OA is to inhibit further inflammation in the joint. In order to demonstrate the anti‐inflammatory actions of EV isolated from BM‐MSC, we investigated the effects of EV on the gene expression of cartilage degrading factors (MMP‐13 and ADAMTS4) and pro‐cartilage factor ACAN in the presence of the inflammatory cytokines IL‐1β or TNF‐α at two different concentrations. Chondrocytes were co‐cultured with EVs such that the number of EVs added to the cells was equivalent to those released from a 1:1 ratio of MSCs to chondrocytes as previously described in a human model^35^ and, as such, the ‘dosage’ or EV was speculative in this experimental model. Further work is required to elucidate the optimum concentration of EV for maximum effect.

Culture in increasing concentrations of IL‐1β or TNF‐α increased MMP‐13 and ADAMTS4 expression in a dose dependant manner as has been reported previously.^36^ The BM‐MSC‐derived EV reduced the inflammatory effects of IL‐1β and TNFα in chondrocytes, significantly decreasing the upregulation of gene expression of both MMP‐13 and ADAMTS4 at certain concentrations, as has been reported. This anti‐catabolic effect is similar to that reported for corticosteroids, a therapeutic agent used clinically to manage equine OA.^36^, ^37^

Whilst these results demonstrate that BM‐MSC EV have anti‐inflammatory effects when chondrocytes are cultured in the presence of IL‐1β or TNF‐α, no effect of culture in the presence of EV on aggrecan gene expression was detected. Despite high levels of IL‐1β causing a large drop in the expression of ACAN gene expression, the application of EV was not able to significantly increase ACAN gene expression. Altered cytokine levels of TNF‐α did not significantly affect ACAN gene expression and nor was a significant effect noted when EV were applied. This may be a preliminary indicator that EV are not a tool for tissue regeneration, although this observation requires further investigation.

One limitation of the study is that the affectof EV on gene expression of MMP‐13, ADAMTS4 and ACAN on unstimulated chondrocytes was not included as a control; furthermore, a control with only PBS and no EV could have been used. Further studies merit inclusion of such control samples in order to help verify the affects of EV on chondrocytes.

This study has demonstrated that equine BM‐MSC produce EV can be isolated by differential sequential ultracentrifugation, that the EV can be internalised into chondrocytes and have anti‐catabolic properties when chondrocytes are cultured in the presence of IL‐1β or TNF‐α.

Whilst MSC have been shown to be efficacious in the treatment of joint disease in the horse,^38^, ^39^ MSC therapy has inherent drawbacks including provoking an immune response. In addition, the practical challenges of removing equine bone marrow and subsequent sterile cellular expansion prior to re‐injection makes MSC therapy prohibitively costly in many cases. The results of this study show that BM‐MSC EV should be investigated further as a possible therapeutic intervention for inflammatory joint disease in the horse.^40^

## ETHICAL STATEMENT

All tissue was taken from cadaveric horses with the owners' full consent.

## AUTHOR CONTRIBUTIONS

William Edward Hotham and Charlotte Thompson equally contributed to the data acquisition. Lin Szu‐Ting is responsible for osteogenic differentiation data acquisition. William Edward Hotham led the manuscript production with assistance from Charlotte Thompson, and Frances Margaret Daphne Henson supervised the work and helped in manuscript editing.

## Data Availability

The data that support the findings of this study are available from the corresponding author upon reasonable request.
